# Network pharmacology and molecular docking to explore the pharmacological mechanism of Yifei Tongluo granules in treating idiopathic pulmonary fibrosis: A review

**DOI:** 10.1097/MD.0000000000033729

**Published:** 2023-06-02

**Authors:** Yuan Hou, Guoyu Wang, Shuo Han, Huaman Liu, Xinhua Jia

**Affiliations:** a Institute of Chinese Medicine Documentation and Culture, Shandong University of Traditional Chinese Medicine, Jinan, Shandong, China; b Beijing Shijitan Hospital Affiliated to Capital University of Medical Sciences, Beijing, China; c China Emergency Department, Zhangdian District Traditional Chinese Medicine Hospital, ZiBo, Shandong, China; d Emergency Department, Zhangdian District Traditional Chinese Medicine Hospital, ZiBo, Shandong, China; e Department of General Medicine, Affiliated Hospital of Shandong University of Traditional Chinese Medicine, Jinan, Shandong, China; f Department of Pulmonary Diseases, Affiliated Hospital of Shandong University of Traditional Chinese Medicine, Jinan, Shandong, China.

**Keywords:** idiopathic pulmonary fibrosis, molecular docking, network pharmacological analysis, Yifei Tongluo granule

## Abstract

Idiopathic pulmonary fibrosis (IPF) is an interstitial lung disease that leads to progressive dyspnea and dry cough, with extracellular matrix deposition as the main pathological feature. Yifei Tongluo granules (YTG) are a traditional Chinese medicine formula that could nourish Qi-Yin, clear phlegm, and invigorate blood circulation. In this research, network pharmacology and molecular docking were used to elucidate the potential mechanism of YTG for treating IPF. A total of 278 biologically active compounds were included in YTG, and 16 compounds were selected for pharmacological analysis and molecular docking through “drugs-compounds-intersecting targets of YTG and IPF” network construction. Protein-protein interaction network was constructed using 330 YTG-IPF intersecting targets. Furthermore, Gene Ontology and Kyoto Encyclopedia of Genes and Genomes pathway enrichment analysis were performed. A total of 10 core targets were screened by protein-protein interaction, and molecular docking was used to further validate the binding ability of the compounds to the core targets. The network pharmacology and molecular docking results showed that Danshenol A, isorhamnetin, Ginsenoside-Rh4, quercetin, and kaempferol might be the main active compounds in the treatment of IPF by YTG, whereas MAPK1, MAPK3, EGFR, and SRC are the core targets while PI3K/AKT pathway and MAPK pathway are the main signaling pathways through which YTG regulates relevant biological processes to intervene in IPF. This study shows that YTG can treat IPF by inhibiting the epithelial-mesenchymal transit process, fibroblast proliferation, fibroblast-to-myofibroblast conversion, myofibroblast anti-apoptosis, collagen expression, and other mechanisms.YTG can be widely used as an adjuvant therapy for IPF in clinical practice, and this study provides the basis for subsequent experimental studies.

## 1. Introduction

Idiopathic pulmonary fibrosis (IPF) is the most common form of chronic idiopathic interstitial pneumonia, with a high incidence and a more insidious onset in elderly individuals. The main clinical manifestations of IPF are progressive dyspnea and dry cough, which seriously affect the quality of life of patients.^[1]^ Its irreversible lung damage results in a high clinical mortality rate, with a median survival of only 2.5 to 3.5 years after diagnosis.^[2]^ In terms of treatment, pirfenidone and nintedanib are approved by the US Food and Drug Administration as drugs with multiple pharmacological mechanisms for the treatment of IPF but with issues of tolerability.^[3]^ Lung transplantation, a treatment that patients less frequently undertake due to the high number of comorbidities in the elderly, postoperative care, and financial issues, is considered the only cure for IPF. As the global aging problem increases, IPF is expected to become a global health issue. Based on the disease characteristics of IPF, finding new therapeutic targets and effective treatment options is essential to slow the progression of or even prevent IPF.

With thousands of years of development and experience, traditional Chinese medicine (TCM) has a complete and rigorous theoretical basis for understanding the etiology of IPF and has also achieved good clinical treatment of this disease. TCM prescriptions have become a routinely used adjunctive therapy in clinical practice. According to the TCM, due to external evil or the patient’s own Qi deficiency, the lungs become dysfunctional, and phlegm and blood stasis bind in the lung. Hence, the TCM pathogenesis of IPF is lung atrophy without enough nourishment. Yifei Tongluo granules (YTG) consists of 9 Chinese medicines, namely, 15 g ginseng, 12 g Ophiopogon Japonicus, 9 g Schisandra chinensis Fructus, 30 g Astragali Radix, 9 g Ginkgo Semen, 3 g leech, 12 g Fritillaria thunbergii, 15 g Mori Cortex, and 15 g Salvia miltiorrhiza. When given this prescription, Qi-Yin can be nourished and phlegm and stasis can be resolved. Although the use of YTG is clinically effective in alleviating symptoms, such as chest tightness, wheezing, and dry cough in patients, its active compounds, possible targets of action, and signaling pathways regulated by it need to be explored further.

The composition of a TCM prescription is complex, and its efficacy results from the combined action of multiple compounds, targets, and pathways. Various compounds may modulate the biological network. Network pharmacology has become an effective research method for investigating the active compounds and the mechanism of pharmacodynamic action of compounds by constructing and resolving the biological network enrichment relationship of drug-target-disease.^[4]^ In this study, we used the network pharmacology approach to collect practical bioactive components and relevant disease targets of YTG for the treatment of IPF. The core proteins were screened by protein-protein interaction (PPI) network analysis, Gene Ontology (GO), and Kyoto Encyclopedia of Genes and Genomes (KEGG) enrichment analysis were used to illustrate the biological functions of the intersecting genes and the cellular pathways involved. The final molecular docking technique verifies the affinity between the screened receptors and ligands. We illustrated the targets and signaling pathways through which YTG inhibits IPF using a network pharmacology approach and molecular docking.

## 2. Methods and materials

### 2.1. Network pharmacology research

#### 1.2.1. Collection of compounds and targets.

The YTG compounds were obtained from the TCM Systems Pharmacology (TCMSP https://old.tcmsp-e.com/tcmsp.php).^[5]^ The screening criteria were presented as follows: oral bioavailability ≥ 30% and drug similarity ≥ 0.18. The SwissADME website (http://www.swissadme.ch/index.php) was used to screen compounds with biological activity contained in ophiopogon japonicus and leech for gastrointestinal absorption. “High” and drug-likeness (drug-like properties) in Lipinski, Ghose, Veber, Egan, and Muegge, 3 or more of the 5 algorithms were selected as “YES.”^[6]^ Next, the standard smile nodes of each compound were obtained using the PubChem (https://pubchem.ncbi.nlm.nih.gov/).^[7]^ Lastly, the standard smile nodes of the identified compounds were uploaded to the SwissTarget (http://www.swisstargetprediction.ch/) database to predict their potential targets.^[8]^ Meanwhile, we searched the OMIM (https://www.omim.org/) database, Gene Cards (https://www.genecards.org/) database, and Therapeutic Target Database (http://db.idrblab.net/ttd/) database with “idiopathic pulmonary fibrosis” for IPF-related disease targets. Also, we integrated and deduplicated those targets obtained from the 3 databases and used the Uniprot (https://www.uniprot.org/) database to standardize the names of the obtained disease targets.^[9]^

#### 2.2.1. Construction of PPIs.

Venny 2.1.0 (http://bioinfogp.cnb.csic.es/tools/venny/) was used to screen common potential therapeutic targets between YTG and IPF-related targets,^[10]^ and the intersected genes were inputted into the STRING database (https://string-db.org/) to obtain the PPI.^[11]^ The PPI network was visualized by Cytoscape 3.9.1 software.^[12]^

#### 3.2.1. GO and KEGG enrichment analysis.

Intersecting targets of YTG and IPF were analyzed for GO and KEGG enrichment using the DAVID2021 platform (https://david.ncifcrf.gov/tools.jsp),^[13]^ an annotated database of biological information, and the results were visualized using bioinformatics (http://www.bioinformatics.com.cn/).

### 2.2. Molecular docking

We obtained 3D structures of compounds (SDF file format) at the PubChem website and converted the SDF into mol2 format using OpenBabel software. The high-resolution 3D structures of the core target protein were downloaded from the PDB database (https://www.rcsb.org/). The water molecules of the core target proteins were removed using PyMOL software. Then, we used Autodock4 software to affix hydrogen atoms to target proteins, and charge calculation and nonpolar hydrogens were merged into their corresponding carbons. The processed receptors were saved in PDBQT format. Compounds were also hydrogenated. Nonpolar hydrogens were merged to their corresponding carbon rotations and set to auto-twist using the AutoDock4 software and then set to a ligand and exported to PDBQT format. The processed ligands and receptors were docked using AutoDock Vina software. If the binding energy is <0 kJ · mol^−1^, then the compound can spontaneously bind to the target protein.^[14]^ We used Discovery Studio software to visualize the docking results.

### 2.3. Ethical approval

The current analysis does not require ethical approval, because our analysis only collects uploaded data information from the public database search. The article does not involve any patient’s personal data and will not cause any patient hurt.

## 3. Results

### 3.1. Network establishment between YTG and IPF

**Figure 1.**
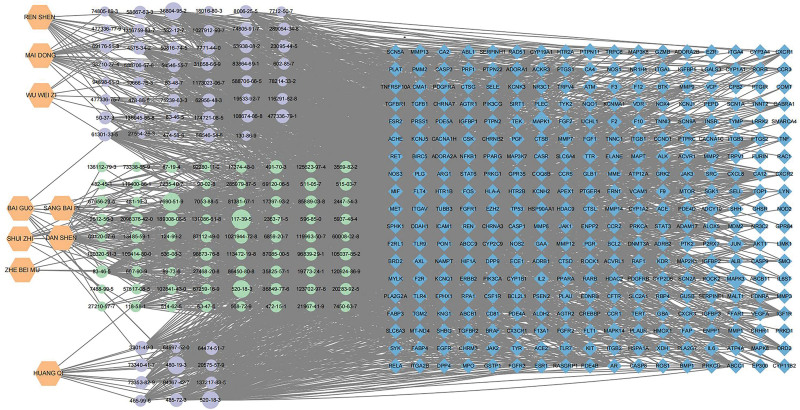
“drugs-compounds-intersecting targets of YTG and IPF” network. The orange hexagon represents 9 kinds of Chinese medicines that make up YTG; the elliptical generation represents 131 compounds screened from 9 Chinese medicines; and the blue diamond represents 330 intersection targets of YTG and IPF. IPF = idiopathic pulmonary fibrosis, YTG = Yifei Tongluo granules.

Through the TCMSP database and literature search, and then condition screening by swissADME, 278 eligible compounds were screened in YTG, that is, 21 for Ginkgo Semen, 65 for Salvia miltiorrhiza, 20 for Astragali Radix, 60 for Ophiopogon Japonicus, 23 for ginseng, 31 for Mori Cortex, 35 for leech, 8 for Schisandrae chinensis Fructus, and 7 for Fritillaria thunbergii. After the removal of duplicate compounds, 131 active compounds were obtained. The target proteins were carried out one by one by Swiss Target Prediction website (www.swisstargetprediction.ch), and the target proteins with a score > 0 were selected according to their probability. After removing the duplicate proteins, 1152 YTG targets were obtained. We searched the OMIM, Gene Cards, and Therapeutic Target Database databases with “Idiopathic pulmonary fibrosis” for IPF-related disease targets, integrated and deduplicated the targets obtained from the 3 databases, and used the UniProt (https://www.uniprot.org/) database to standardize the names of the acquired disease targets. A total of 1752 IPF-related targets were obtained. Venny 2.1.0 (http://www.liuxiaoyuyuan.cn/) was used to screen targets between YTG and IPF-related targets. A total of 330 intersected targets were obtained. The network of “drugs-compounds-intersecting targets of YTG and IPF” was analyzed using Cytoscape 3.9.1 software (Fig. 1), and each edge indicates the compounds and the interaction between the compound and the target. The top 16 compounds and their structural information were ranked according to the degree ranking (Table 1).

**Table 1:** Basic information on the main active compounds of YTG.

CAS	Molecule name	OB%	DL	Herbs
117-39-5	Quercetin	46.43	0.28	Ginkgo Semen astragali Radix mori cortex
7690-51-9	Pelargonidin	37.99	0.21	Mori cortex fritillariae thunbrgii bulbus
480-19-3	Isorhamnetin	49.60	0.31	Ginkgo Semen astragali Radix
520-18-3	Kaempferol	41.88	0.24	Ginkgo Semen astragali Radix mori cortex ginseng
36804-95-2	Deoxyharringtonine	39.27	0.81	Ginseng schisandrae chinensis fructus
477336-79-1	Ophiopogonanone F	--	--	Ophiopogon japonicus
62956-48-3	Gomisin G	32.68	0.38	Schisandrae chinensis fructus
1021944-72-8	Hirudinoidine A	–	–	Leech
485-72-3	Formononetin	69.67	0.21	Ginkgo Semen astragali Radix
82078-76-0	Schizandrer B	30.71	0.83	Schisandrae Chinensis Fructus
3301-49-3	Kumatakenin	50.83	0.29	astragali Radix
61301-33-5	Schisandrin C	46.27	0.84	Schisandrae Chinensis Fructus
189308-08-5	Danshenol A	56.97	0.52	Radix Salviae
511-05-7	Sugiol	36.11	0.28	Radix Salviae
83-46-5	Beta-Sitosterol	36.91	0.75	Ginkgo Semen Ginseng mori cortex fritillariae thunbrgii bulbus
174721-08-5	Ginsenoside-Rh4	31.11	0.78	Ginseng

YTG = Yifei Tongluo granules.

### 3.2. PPI network

**Figure 2.**
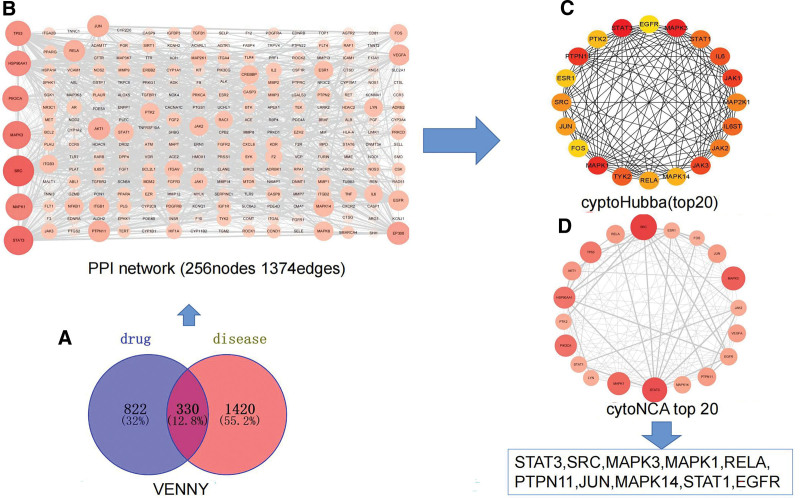
Network and core targets of YTG in the treatment of IPF. (A) Venn diagram of the target of YTG and the target of IPF. (B) The PPI network of YTG and IPF targets. Nodes represent proteins (the colors from pink to red represent the degree of binding between proteins). Edge represents protein-protein association. (C) Topological analysis based on CytoHubba, the yellow to red represents the degree value of the target in the PPI network. (D) Centrality analysis based on cytoNCA. IPF = idiopathic pulmonary fibrosis, PPI = protein-protein interaction, YTG = Yifei Tongluo granules.

To further identify the core targets, 330 YTG-IPF-related targets were entered into the STRING (https://cn.string-db.org/) database, wherein the species was limited to “Homo sapiens,” the minimum required interaction score was set to 0.9, and the independent target protein nodes were hidden. A PPI network with 330 nodes, 6782 edges, an average node degree of 41.1, and a PPI enrichment *P* value: <1.0^e−6^ was obtained (Fig. 2). CytoNCA, a plug-in Cytoscape software, was used to perform the centrality analysis and evaluation of PI networks. In CytoNCA, betweenness centrality, closeness centrality (CC), degree centrality, and network centrality were selected for the analysis, and the higher the parameter value is, the more important the target is. Meanwhile, the MCC calculation method in the CytoHubba plug is selected for topology analysis. Ultimately, STAT3, SRC, MAPK3, MAPK1, RELA, PTPN11, JUN, MAPK14, STAT1, and EGFR were identified as the core targets of YTG for the treatment of IPF (Table 2).

**Table 2:** Target proteins with potentially critical roles in YTG treatment of IPF.

Targets	Degree centrality	Betweenness centrality	Closeness centrality	Network centrality
STAT3	62	4371.943	0.484556	40.851818
SRC	67	8510.347	0.502	42.64326
MAPK3	58	2810.5413	0.47718632	37.467506
MAPK1	55	2492.148	0.47358492	33.95374
RELA	39	1125.8406	0.4466192	21.84547
PTPN11	39	755.6746	0.41973245	22.602667
JUN	39	1676.1593	0.4482143	21.051645
MAPK14	35	2582.1265	0.4403509	15.49445
STAT1	32	541.20734	0.43425605	16.68624
EGFR	35	1352.9712	0.43881118	16.586817

IPF = idiopathic pulmonary fibrosis, YTG = Yifei Tongluo granules.

### 3.3. GO and KEGG analysis

**Figure 3.**
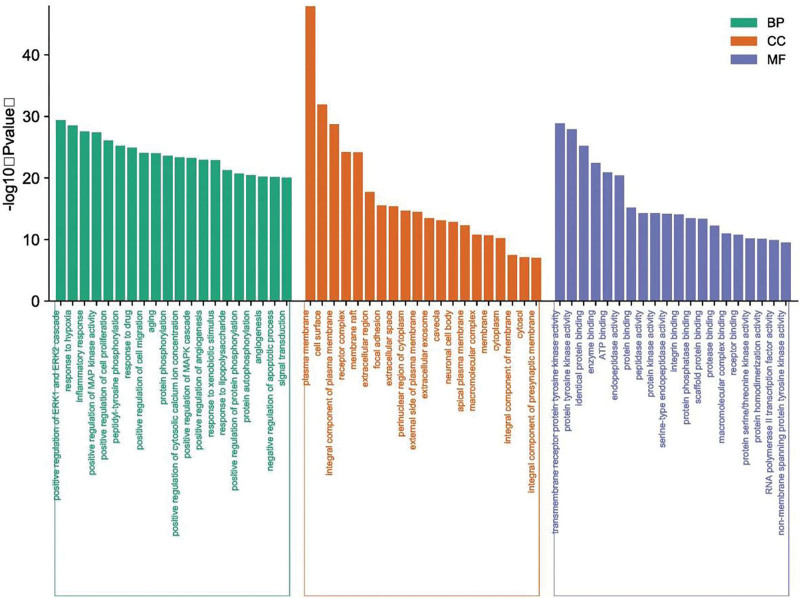
GO enrichment analysis of targets of YTG in treating IPF (TOP20). GO = Gene Ontology, IPF = idiopathic pulmonary fibrosis, YTG = Yifei Tongluo granules.

We used the DAVID 6.8 database for GO and pathway enrichment analysis of 330 YTG-IPF-related genes, limiting the species to Homo sapiens, and obtained 899 statistically significant (*P* < .01) GO function enrichment entries, including 669 biological process (BP), 141 molecular functions (MF), and 89 CC terms in total. GO terms were sorted by -log10 (*P* value), and the top 20 terms in BP, CC, and MF were selected and plotted in the column chart (Fig. 3). The GO analysis results indicate that YTG may be involved in BP through the positive regulation of ERK1 and ERK2 cascade, inflammatory response, cell proliferation, cell migration, peptidyl-tyrosine phosphorylation, and protein phosphorylation, etc. In terms of MF, it mainly contains plasma membrane, cell surface, integral compound of plasma membrane, receptor complex, membrane raft, extracellular region, focal adhesion, and extracellular space. In terms of cellular composition, the main compounds are plasma membrane, cell surface, integral compound of the plasma membrane, receptor complex, membrane raft, extracellular region, and focal adhesion. In terms of MF, the main functional processes involved are transmembrane receptor protein tyrosine kinase activity, protein tyrosine kinase activity, identical protein binding, enzyme binding, and ATP binding.

**Figure 4.**
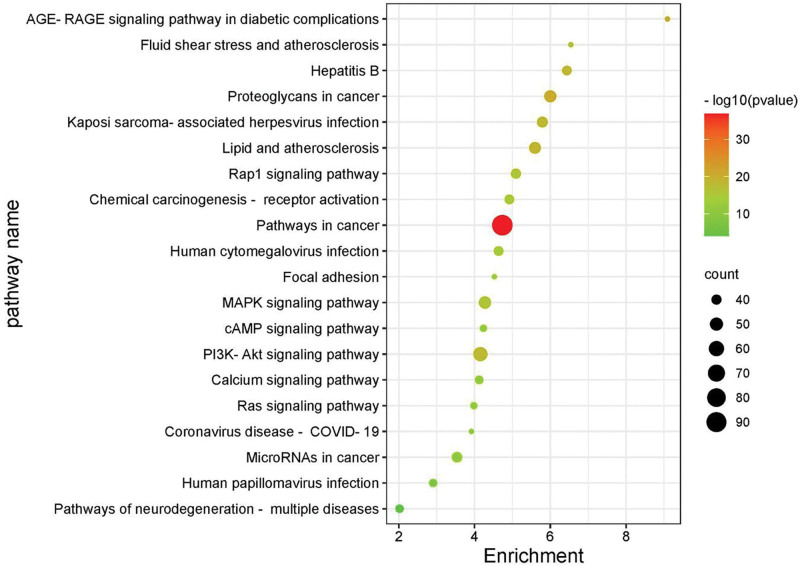
KEGG pathway enrichment analysis of targets of YTG in treating IPF (top 20). IPF = idiopathic pulmonary fibrosis, KEGG = Kyoto Encyclopedia of Genes and Genomes, YTG = Yifei Tongluo granules.

A total of 148 YTG-IPF-related pathways were screened by KEGG enrichment analysis (*P* < .01). The top 20 potential signaling pathways with the highest gene counts are presented as bubble plots in Figure 4. The bubble area represents the number of enriched genes in the pathway. The bubble color represents the size of the *P* value, and the bluer the bubble color means higher significance of the signaling pathway. The 330 intersection genes were mainly involved in KEGG pathways, including tumor pathway, PIK3-AKT signaling pathway, MAPK signaling pathway, proteoglycans in cancer, lipids and atherosclerosis, and Kaposi sarcoma-associated herpesvirus infection. The GO and KEGG analysis results indicated that YTG might regulate the PI3K-AKT signaling pathway and MAPK signaling pathway to interfere with apoptosis, cell proliferation, and inflammatory response to treat IPF.

### 3.4. Molecular docking verification

**Figure 5.**
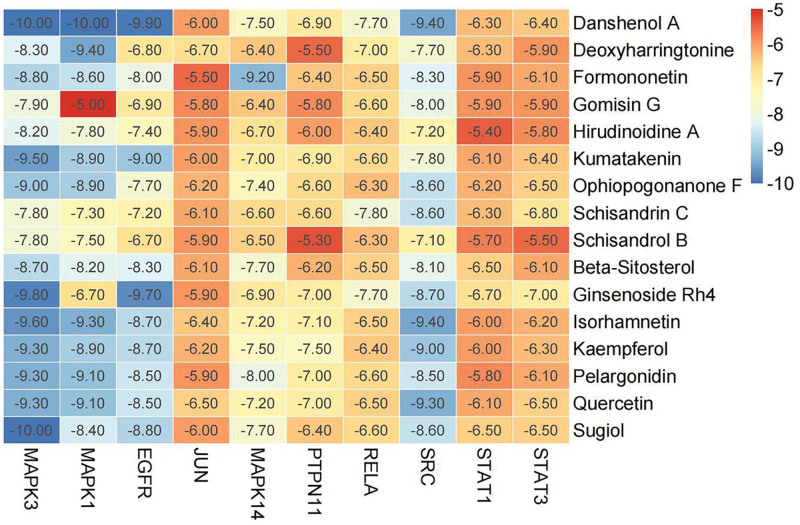
Molecular docking heat diagram of 16 compounds and 10 core targets.

**Figure 6.**
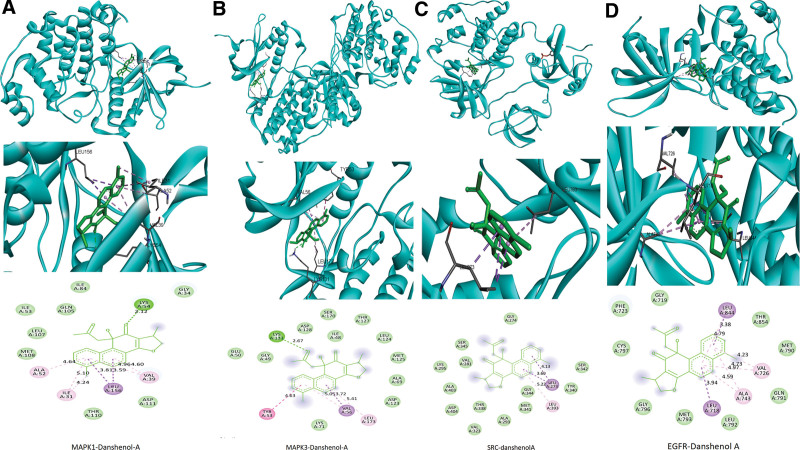
Molecular docking results of Danshenol A bound to 4 core targets. (A) MAPK1-Danshenol A; (B) MAPK3-Danshenol A; (C) SRC-Danshenol A; (D) EGFR-Danshenol A.

Based on the preliminary network pharmacology results, we used the 16 screened high-degree compounds to molecularly dock to 10 core targets. The ligand’s binding energy to the receptor is shown in Figure 5. The molecular docking results showed that the binding energies of all 16 compounds with each of the 10 core targets were <−5 kcal/mol, indicating that the core compounds of YTG could effectively bind to the core targets of YTG-IPF to exert their drug effects. Further analysis showed that the docking activity of the 4 core targets, namely, MAPK3, MAPK1, EGFR, and SRC, was significant. Table 3 lists the bonding energy of each of these 4 core targets with the top 5 compounds, whereas Table 3 reveals that Danshenol A (DA) has a high affinity for MAPK3, MAPK1, EGFR, and SRC. Figure 6 depicts a visual image of DA bound to these 4 core targets. Figure 6A shows DA forming a hydrogen bond at LYS54, a pi-sigma bond at LEU156, 2 pi-Alkyl bonds at ILE31 and YAL39, and a pi-Alkyl bond at ALA52 with MAPK1. Figure 6B shows that DA binds to MAPK3 by hydrogen bonding, pi-pi Stacked, pi-sigma, and pi-Alkyl interactions with LYS131, TYR53, LYS71, VAL56, and LEU173, respectively. Figure 6C shows DA binding in the docking pocket of SRC by forming 2 pi-sigma bonds at LEU273 and 1 pi-Alkyl bond at LEU393. Figure 6D shows DA forming pi-sigma bonds in the EGFR docking pocket in LEU718, LEU844 1 each, and 2 pi-Alkyl bonds in ALA743 and VAL726.

**Table 3:**
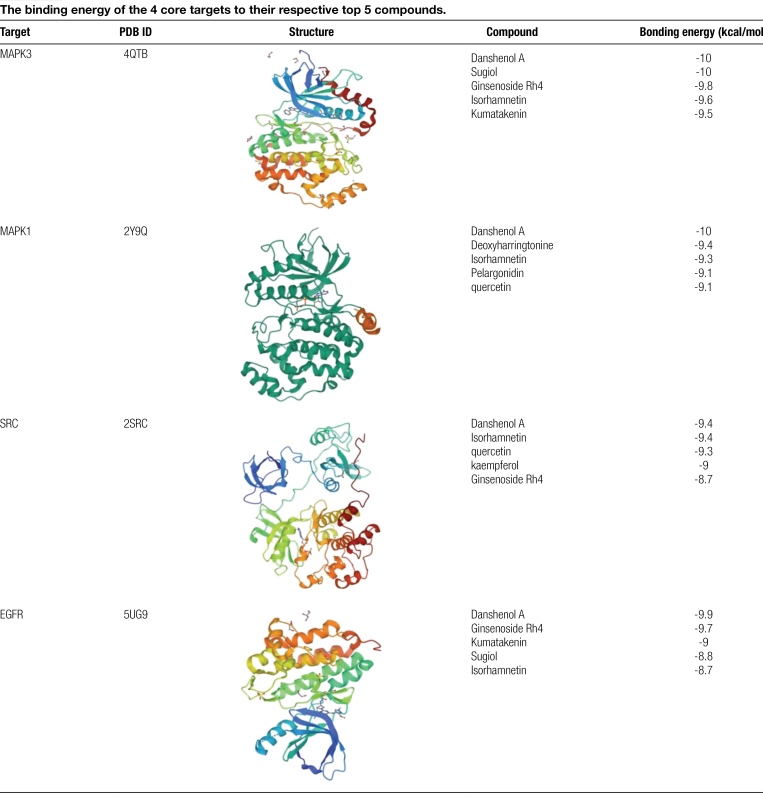
The binding energy of the 4 core targets to their respective top 5 compounds.

## 4. Discussion

IPF is an unreversible, chronic progressive disease with typical histopathological manifestations, such as fibroblast foci, epithelial cell proliferation, and honeycomb cysts.^[1]^ Patients often suffer from dyspnea, such as chest tightness and breathlessness, which seriously affects their quality of life. Thus, slowing down or even reversing the pathological process of pulmonary fibrosis has become an urgent clinical problem. In this study, we used a network pharmacology approach to explore the pharmacological mechanisms of YTG in treating IPF by constructing a “drugs-compounds-Intersecting targets of YTG and IPF” network. Then we used the molecular docking method to predict the binding ability of the core compounds and core targets obtained from the network pharmacology analysis. Drugs-compounds-intersecting targets of YTG and IPF.

YTG, as a typical TCM prescription with rigorous formulation and logical grouping, consists of 9 Chinese TCMs that contain a wide variety of compounds, and the biological activities and pharmacological effects of various compounds are more complex. We screened for compounds with pharmacokinetic activity and good absorption and distribution in the human body. A total of 278 compounds were identified from 9 TCMs. By constructing a “drugs-compounds-intersecting targets of YTG and IPF” network for YTG, we identified the core active ingredients of YTG, namely, DA, isorhamnetin, Ginsenoside-Rh4, quercetin, and kaempferol. The antifibrotic effects of quercetin and kaempferol have been extensively studied. The main target of kaempferol is TGF-β, which inhibits the epithelial-mesenchymal transit (EMT) process by reversing the expression of E-cadherin and α-SMA. In addition, kaempferol may act as an antifibrotic agent by regulating autophagy and inhibiting the expression of inflammatory factors.^[15]^ Quercetin inhibits the lung fibrosis process through a variety of pathways. For example, quercetin blocks the SphK1/S1P signaling pathway during fibroblast activation; it also promotes FasL receptor and caveolin-1 expression and inhibits AKT activation of the apoptotic program in senescent fibroblasts.^[16,17]^ Various components of Danshen have antifibrotic effects.^[18,19]^ DA and isorhamnetin are all present in Salvia miltiorrhiza. Isorhamnetin is a natural antioxidant that hinders the EMT process and endoplasmic reticulum stress by modulating the PERK signaling pathway to reduce bleomycin-induced pulmonary fibrosis in mice.^[20,21]^ DA is an abietane-type diterpene ester derived from the roots of Salvia miltiorrhiza. Although the biological effects of DA have not been studied as extensively as those of other tanshinones, evidence shows that DA inhibits the expression of tumor necrosis factor-α, interleukin-1β, and interleukin-8 at the gene level and exhibits superiority over the anti-inflammatory activity of tanshinone IIA.^[22]^ DA also inhibits tumor necrosis factor-α-induced reactive oxygen species production and suppresses ICAM-1-mediated inflammatory cell adhesion and aggregation in vascular endothelial cells.^[23]^ DA ameliorates myocardial fibrosis by inhibiting mitochondrial dysfunction and reducing reactive oxygen species production.^[24]^ The biological effects exhibited by these compounds suggest that YTG may act as an antifibrotic agent through multiple mechanisms.

Through PPI network construction, we predicted that the core biological targets of YTG for IPF treatment were STAT3, SRC, MAPK3, MAPK1, RELA, PTPN11, JUN, MAPK14, STAT1, and EGFR. Among them, EGFR, MAPK3, MAPK1, and SRC showed the best binding energy with our previous YTG-screened compounds. EGFR is an epidermal growth factor receptor that binds to the receptor as a ligand. It activates the PI3K-AKT signaling pathway and MEK/ERK signaling pathway to transmit extracellular signals into the cell.^[25]^ EGFR levels were abnormally elevated in IPF fibroblast lesions and fibroblasts isolated from IPF lungs, and nintedanib blocked EGFR activation in a concentration-dependent manner to exert an antifibrotic effect on lung fibrosis.^[3]^ SRC, a nonreceptor tyrosine kinase, could be activated by binding to pro-fibrotic cytokines (e.g., transforming growth factor-beta l [TGF-β1], PDGF) and leads to IPF by inducing inflammatory responses, fibroblast differentiation, and EMT.^[26]^ EGFR and SRC play critical roles in regulating cell BPs, such as IPF-related epithelial cell apoptosis, fibroblast proliferation and differentiation, and epithelial cell migration.^[25]^ As discussed earlier, EGFR binding to receptor tyrosine kinases activates the MEK-ERK signaling cascade, and MAPK1 (ERK2) and MAPK3 (ERK1) are essential compounds of the Ras-Raf-MEK-ERK signaling cascade.^[27]^ Animal experiments show that TGF-β1 leads to fibroblast-myofibroblast differentiation in mice by increasing the expression of MEK1/2 and ERK1/2 phosphorylation.^[28]^ The mouse MEK-specific blocker ARRY selectively inhibits TGF-a-induced phosphorylation of ERK1/2 in fibroblasts, inhibits fibroblast proliferation, and improves lung compliance to achieve antipulmonary fibrosis effects.^[29]^

To illustrate the functional and signaling pathways of the 330 protein targets associated with YTG for IPF, we performed GO and KEGG analyses. The results showed that the target genes were mainly enriched in the positive regulation of ERK1 and ERK2 cascade, inflammatory response, response to hypoxia, positive regulation of MAP kinase activity, positive regulation of cell proliferation and cell migration, and other biological pathways. KEGG analysis showed that 330 target genes were enriched in 148 signaling pathways, indicating that YTG acts as a treatment for IPF by regulating multiple signaling pathways. Among them, the PI3K-AKT pathway and MAPK pathway showed high enrichment. In recent years, IPF can be viewed as a proliferative lung disease with similar genetic alterations, pathogenic mechanisms, and pathological features between IPF and lung cancer, particularly nonsmall cell carcinoma, and even in treatment strategies.^[30,31]^ Epidermal growth factor receptors, such as EGFR, regulate tumor cell proliferation, apoptosis, angiogenesis, and lymphangiogenesis in small cell carcinoma of the lung mainly through the PI3K-AKT pathway and the Ras/Raf/Mek pathway. At the same time, these 2 pathways also play an essential role in regulating the proliferation and apoptosis of IPF fibroblasts and EMT.^[31,32]^ The study confirms that the Ras-ERK pathway inhibits the TGF-β1-induced EMT process in mouse lung epithelial type-II cells.^[33]^ In human lung epithelial type-II cells, TGF-β1 activates the EGFR-RAS-ERK signaling pathway through the transcriptional regulator zeb1, which leads to fibroblast activation.^[34]^ Satish K. Madala et al^[35]^ demonstrate that the MEK inhibitor ARRY improved lung fibrosis in TGF-a mice by inhibiting lung mesenchymal cell activation without affecting the PI3K-AKT signaling pathway. Studies have shown that the upregulation of the PI3K-AKT signaling pathway leads to mTOR activation and exacerbates bleomycin-induced pulmonary fibrosis when lung epithelial cell autophagy levels fail to inhibit apoptosis.^[29]^ In addition, AKT inhibitors effectively inhibit fibroblast differentiation in lung tissue and reduce collagen I and III levels.^[36]^ This finding suggests that YTG may achieve antifibrotic effects through combined signaling pathway inhibition.

We used molecular docking to predict the binding activity of the bioactive components of YTG to the target proteins. The results showed that the binding energies of our 10 preselected target proteins to 16 bioactive components were all <−5 kcal/mol. Among them, MAPK3-DA, MAPK1-DA, and MAPK3-Sugiol showed the lowest binding energy (−10 kcal/mol), indicating that these 3 receptor-ligand pairs were the most stable in binding. Based on this finding, we suggest that DA is the most prominent compound in YTG, and MPAK3 MAPK1 is the most critical target of YTG for treating IPF.

## 5. Conclusion

In this study, we used a network pharmacology and molecular docking approach to predict the potential mechanism of YTG for treating IPF. The results showed that DA, isorhamnetin, quercetin, and kaempferol were the main active compounds in YTG. Meanwhile, MAPK3, MAPK1, EGFR, and SRC are the potential therapeutic targets of YTG for treating IPF. YTG treats IPF by regulating the PI3K-AKT signaling pathway, MAPK signaling pathway, and other pathways that inhibit the EMT process, fibroblast proliferation, fibroblast-to-myofibroblast cell conversion, apoptosis resistance of myofibroblasts, collagen expression, and other mechanisms. Our study provides a reference for further research into the molecular mechanisms of YTG in the treatment of IPF.

### 5.1. Strengths and limitations of this study

However, this study has some limitations. First, the TCMSP was used to identify the compounds in YTG. Still, probably not all chemical compounds with biological activity were included, and compounds with unproven physical activity were excluded. Thus, the accuracy and timeliness of the database need to be further verified. Second, we identified the essential active compounds of YTG through a network pharmacological approach. Still, these compounds only do not account for the complete mechanism and pathway of action of YTG in treating IPF and only serve as supporting evidence for the efficacy of YTG. Finally, the effects of target proteins identified through the pharmacology network approach in the ponderous and sophisticated human system have not been fully explored and explained. Based on the experimental validation, we believe that YTG has excellent research potential in antipulmonary fibrosis, and the targets and pathways of action of the vital active compounds of YTG are worthy of further study and exploration.

## Author contributions

**Conceptualization:** Xinhua Jia.

**Data curation:** Yuan Hou, Guoyu Wang, Shuo Han, Xinhua Jia.

**Investigation:** Guoyu Wang.

**Methodology:** Guoyu Wang.

**Software:** Shuo Han, Xinhua Jia.

**Supervision:** Huaman Liu.**Validation:** Yuan Hou.

**Visualization:** Yuan Hou, Shuo Han, Huaman Liu, Xinhua Jia.

**Writing – original draft:** Yuan Hou.

**Writing – review & editing:** Huaman Liu.
